# Altered cortical functional network during behavioral inhibition in individuals with childhood trauma

**DOI:** 10.1038/s41598-018-28329-6

**Published:** 2018-07-04

**Authors:** Sungkean Kim, Ji Sun Kim, Miseon Shim, Chang-Hwan Im, Seung-Hwan Lee

**Affiliations:** 1 Clinical Emotion and Cognition Research Laboratory, Inje University, Goyang, Republic of Korea; 2 Department of Biomedical Engineering, Hanyang University, Seoul, Republic of Korea; 3 Department of Psychiatry, Soonchunhyang University Cheonan Hospital, Cheonan, Republic of Korea; 4 Department of Psychiatry, University of Missouri-Kansas City, Kansas City, Missouri USA; 5 Department of Psychiatry, Inje University, Ilsan-Paik Hospital, Goyang, Republic of Korea

## Abstract

Individuals who have prior history of childhood traumatic experiences are at a high risk for a variety of psychological and behavioral problems throughout their lifetime. This study aimed to investigate whether such individuals exhibit altered cortical functional networks during a behavioral inhibition task. One hundred fifty-three non-clinical individuals were recruited and instructed to perform a Go/NoGo task during an electroencephalograph. Source-level weighted functional networks based on the graph theory were analyzed for NoGo-P3 processing. Based on their total scores on the childhood trauma questionnaire (CTQ) participants were divided into three groups: low CTQ, middle CTQ, and high CTQ. Results at the global level indicated decreased strength, clustering coefficient, and efficiency for the low and gamma bands in the high CTQ group. In addition, the path length of the low beta band was observed to be longer in the high CTQ group than the low CTQ group. At the nodal level, the nodal clustering coefficient of high CTQ group was decreased in left primary somatosensory cortex and middle occipital gyrus for the low beta band, and in left superior temporal gyrus for the gamma band. The nodal clustering coefficient of the left primary somatosensory cortex showed a significant negative correlation with the total CTQ score for the low beta band. In addition, the nodal clustering coefficient of the left middle occipital gyrus for the low beta band and superior temporal gyrus for the gamma band showed significant negative correlations with the emotional neglect score. Our results demonstrate an altered cortical functional network in individuals who experienced childhood trauma. In particular, the left primary somatosensory cortex, middle occipital gyrus, and superior temporal gyrus were found to be vulnerable in individuals who experienced childhood trauma, especially emotional neglect.

## Introduction

Childhood trauma appears to be a crucial etiological factor in the development of various psychological and behavioral disorders across the lifespan^1^. In particular, childhood trauma has been associated with multiple adverse cognitive consequences, such as low IQ, decreased academic performance, and deficits in attention, emotion, and inhibitory functioning^2^.

Epidemiological studies reveal that children with early traumatic experiences are more susceptible to developing depression and/or anxiety disorders^3^. Childhood trauma may also play a role in the development of impulsivity that progress into substance abuse and suicidal behaviors^4,5^. Moreover, those with a history of childhood trauma demonstrate impaired behavioral inhibition^6,7^, which is an essential regulatory function that develops progressively from child- to adulthood^8^. Deficits of inhibitory control have been consistently observed in adults who experienced childhood sexual abuse^9^, adolescents with early life stress^10^, and maltreated and institutionalized children^11–13^. In addition, childhood adversity can alter reward processes central to the Behavioral Activation System. Studies have identified an insensitivity to rewards in maltreated children^14^, and less brain activation in regions associated with reward processes during the anticipation of reward in those with childhood trauma^15,16^.

Furthermore, childhood trauma has been associated with cognitive dysfunctions^2,17^, including attentional deficits in both children and adults^7^. Considering that inattention is one of the fundamental symptoms of attention deficit hyperactivity disorder (ADHD)^18^, it is arguable that the exposure to traumatic events during childhood is a risk factor for ADHD in childhood^19^ and later in adulthood^20^.

Meanwhile, the Go/NoGo task has been widely used to explore behavioral inhibition^21,22^. In the Go/Nogo task, individuals are instructed to respond to Go trials and to refrain responding (inhibit) to Nogo trials^21^. Go/NoGo event-related potentials (ERP) have been used as an informative tool to evaluate behavioral inhibitory capacities^23^. Typically, N2 and P3 components of ERPs are analyzed in the Go/NoGo task. These two components generally appear in sequence and are associated with the early and late phases of response inhibition, respectively^24^. NoGo-N2 has been suggested to reflect response inhibition^25^ or the process of conflict monitoring^26^, whereas NoGo-P3 has been proposed to primarily reflect the inhibitory process itself^27^. Recent studies have suggested that NoGo-P3 may play an important role in the post-response stage, reflecting processes of evaluation or monitoring of inhibition, error detection, and preparation for future trials^28,29^.

Childhood traumatic experiences may lead to long-term structural and functional changes in brain development^30–33^. An increasing number of researchers have started to focus on changes in the cortical functional network. Many of these studies have adopted the graph theory to quantify global and local changes in the cortical functional network using network indices such as the strength, clustering coefficient, path length, and efficiency^34–37^. In addition, different modalities, including the resting-state functional network using functional magnetic resonance imaging (fMRI)^38^, the structural cortical network based on cortical thickness^39^, and the structural connectivity network based on diffusion tensor imaging (DTI) tractography^40^ have reported altered brain networks in individuals with childhood trauma. However, no study has evaluated the cortical functional network using electroencephalography (EEG). EEG has significant advantages such as advanced time resolution for investigating the functional network of early neural processing (i.e., the N2 or P3 component) during the Go/NoGo task and cost-effectiveness. While EEG has some limitations such as low spatial resolution that originates from volume conduction^41,42^, and poor signal-to-noise ratios affected by various noises and artifacts^43,44^, they can be addressed through source-imaging methods. In particular, the spatial resolution of EEG can be substantially improved by mapping the scalp potential distribution onto the underlying cortical source space using source-imaging methods.

In the present study, brain cortical networks were evaluated using a source-level weighted functional network analysis during a Go/NoGo paradigm in individuals who had experienced childhood trauma. Source-level weighted network analysis allows observation of alternations in specific local cortical regions as well as in the patterns of the global brain network. We hypothesized that during a behavioral inhibition task, individuals with traumatic childhood experiences would exhibit an altered cortical functional network at both global and nodal levels, reflecting a loss of inhibitory control during a behavioral inhibition task. Furthermore, we hypothesized that the altered cortical network indices such as the strength, clustering coefficient, path length, and efficiency would be significantly correlated with the severity of childhood trauma.

## Results

### Psychological and Behavioral measures

Table 1 shows the comparison of demographic and psychological characteristics between the low, middle, and high CTQ groups. The scores of the STAI, BDI, BIS (attentional impulsivity and motor impulsivity), CAARS (inattention/memory and hyperactivity/restlessness), and CTQ (physical, emotional, sexual abuse, physical, and emotional neglect) were significantly higher in the high CTQ group than in the low CTQ group (SAI: 32.45 ± 7.26 *vs*. 41.89 ± 7.93, *p* < 0.001; TAI: 34.27 ± 9.88 *vs*. 45.87 ± 9.11, *p* < 0.001; BDI: 5.39 ± 3.50 *vs*. 12.08 ± 7.68, *p* < 0.001; attentional impulsivity: 15.39 ± 3.21 *vs*. 17.66 ± 3.72, *p* = 0.009; motor impulsivity: 23.91 ± 4.98 *vs*. 26.42 ± 4.32, *p* = 0.044; inattention/memory: 22.20 ± 4.95 *vs*. 28.86 ± 6.30, *p* < 0.001; hyperactivity/restlessness: 16.84 ± 4.11 *vs*. 20.79 ± 4.62, *p* < 0.001). In contrast, the score of the Behavioral Activation System (drive) was significantly lower in the high CTQ group than in the low CTQ group (9.02 ± 1.91 *vs*. 7.79 ± 1.76, *p* = 0.003). However, there was no significant difference among the three groups in the NoGo false alarm rate (0.12 ± 0.11 *vs*. 0.12 ± 0.08 *vs*. 0.13 ± 0.10, *p* = 0.888). In addition, the three groups did not significantly differ in their reaction time and hit rate during the Go condition (Go reaction time: 373.78 ± 26.53 *vs*. 374.53 ± 25.00 *vs*. 381.25 ± 25.76, *p* = 0.343; Go hit rate: 0.95 ± 0.07 *vs*. 0.94 ± 0.08 *vs*. 0.92 ± 0.07, *p* = 0.116).

**Table 1:** Comparison of baseline demographic, psychological, and behavioral characteristics in individuals with low, middle, and high childhood trauma questionnaire (CTQ) scores.

	Low CTQ (N = 44)	Middle CTQ (N = 71)	High CTQ (N = 38)	*p*	Pairwise test, *p*^a^
	*Mean* ± *SD* or N (%)				Low vs. High
Age (years)	26.14 ± 5.92	28.48 ± 6.74	28.24 ± 6.21	0.142	
Sex					
Male (%)	20 (45.5)	24 (33.8)	12 (31.6)	0.343	
Female (%)	24 (54.5)	47 (66.2)	26 (68.4)		
Education (years)	13.91 ± 1.88	14.56 ± 1.75	14.58 ± 1.54	0.109	
Go reaction time (ms)	373.78 ± 26.53	374.53 ± 25.00	381.25 ± 25.76	0.343	
Go hit rate	0.95 ± 0.07	0.94 ± 0.08	0.92 ± 0.07	0.116	
Nogo false alarm rate	0.12 ± 0.11	0.12 ± 0.08	0.13 ± 0.11	0.888	
State Anxiety Inventory (SAI)	32.45 ± 7.26	36.00 ± 6.97	41.89 ± 7.93	**<0**.**001**	**<0**.**001**
Trait Anxiety Inventory (TAI)	34.27 ± 9.88	39.17 ± 8.23	45.87 ± 9.11	**<0**.**001**	**<0**.**001**
Beck Depression Inventory (BDI)	5.39 ± 3.50	6.92 ± 4.37	12.08 ± 7.68	**<0**.**001**	**<0**.**001**
Barratt Impulsivity Scale (BIS)	58.07 ± 10.17	58.83 ± 8.67	62.95 ± 9.29	**0**.**039**	0.056
Attentional impulsivity	15.39 ± 3.21	15.77 ± 3.36	17.66 ± 3.72	**0**.**006**	**0**.**009**
Motor impulsivity	23.91 ± 4.98	25.28 ± 4.49	26.42 ± 4.32	**0**.**049**	**0**.**044**
Non-planning impulsivity	18.77 ± 4.19	17.77 ± 3.47	18.87 ± 3.83	0.236	
Conners’ Adult ADHD rating scale (CAARS)	70.68 ± 12.08	73.25 ± 15.38	82.87 ± 13.35	**<0**.**001**	**<0**.**001**
Inattention/Memory	22.20 ± 4.95	23.92 ± 6.59	28.86 ± 6.30	**<0**.**001**	**<0**.**001**
Hyperactivity/Restlessness	16.84 ± 4.11	17.86 ± 4.69	20.79 ± 4.62	**<0**.**001**	**<0**.**001**
Impulsivity/Emotional lability	17.27 ± 3.74	17.93 ± 4.91	19.47 ± 3.97	0.070	
Problem with self-concept	14.36 ± 3.14	13.55 ± 2.93	13.95 ± 3.00	0.368	
Behavioral Inhibition System	21.34 ± 2.80	21.08 ± 2.22	21.53 ± 1.69	0.612	
Behavioral Activation System	37.73 ± 7.81	34.85 ± 5.26	34.42 ± 3.92	**0**.**016**	**0**.**035**
Drive	9.02 ± 1.91	8.30 ± 1.36	7.79 ± 1.76	**0**.**003**	**0**.**003**
Fun-Seeking	10.57 ± 2.63	10.28 ± 1.92	10.63 ± 1.94	0.685	
Reward Responsiveness	18.14 ± 7.24	16.27 ± 4.81	16.00 ± 1.79	0.104	
Childhood Trauma Questionnaire	31.41 ± 2.06	40.44 ± 3.61	60.21 ± 10.00	**<0**.**001**	**<0**.**001**
Physical abuse	5.66 ± 1.10	6.54 ± 1.82	10.18 ± 3.91	**<0**.**001**	**<0**.**001**
Emotional abuse	5.07 ± 0.26	5.82 ± 1.36	9.18 ± 3.73	**<0**.**001**	**<0**.**001**
Sexual abuse	5.16 ± 0.65	5.41 ± 0.90	7.61 ± 3.40	**<0**.**001**	**<0**.**001**
Physical neglect	5.55 ± 1.25	6.31 ± 1.91	7.89 ± 2.99	**<0**.**001**	**<0**.**001**
Emotional neglect	9.98 ± 1.89	16.37 ± 3.87	25.34 ± 4.53	**<0**.**001**	**<0**.**001**

^a^*p*-values represent statistically significant differences between the low and high CTQ groups with post-hoc test using Bonferroni correction.

### Global level differences in cortical functional networks

Table 2 shows the comparison of global level indices, including the strength, clustering coefficient, path length, and efficiency of each frequency band among the low, middle, and high CTQ groups. The strength, clustering coefficient, and efficiency of the low beta and gamma bands were significantly decreased in the high CTQ group compared to the low CTQ group (strength: 179.14 ± 10.79 *vs*. 173.50 ± 7.60, *p* = 0.001; clustering coefficient: 0.57 ± 0.03 *vs*. 0.55 ± 0.02, *p* = 0.001; efficiency: 0.57 ± 0.03 *vs*. 0.55 ± 0.02, *p* = 0.001 for low beta band, strength: 134.70 ± 18.02 *vs*. 126.69 ± 12.83, *p* = 0.002; clustering coefficient: 0.42 ± 0.06 *vs*. 0.39 ± 0.04, *p* = 0.002; efficiency: 0.43 ± 0.06 *vs*. 0.40 ± 0.04, *p* = 0.002 for gamma band). On the other hand, the path length of the low beta band was significantly longer in the high CTQ group than in the low CTQ group (1.85 ± 0.09 *vs*. 1.90 ± 0.07, *p* = 0.002). However, there was no significant difference among the three groups in other frequency bands. The power was 0.95 to detect an effect size of 0.13 in comparison of the cortical network characteristics at the global level among the three groups.

**Table 2:** Mean and standard deviation values of global network indices including strength, clustering coefficient, path length, and efficiency in each frequency band among low, middle, and high childhood trauma questionnaire (CTQ) groups.

	Low CTQ (N = 44)	Middle CTQ (N = 71)	High CTQ (N = 38)	Effect size (η^2^)	*p*	Pairwise test, *p*^a^
	*Mean* ± *SD*					Low vs. High
Alpha band						
Strength	208.09 ± 9.51	205.74 ± 8.98	205.56 ± 8.74	0.027	0.139	
Clustering coefficient	0.66 ± 0.03	0.65 ± 0.03	0.65 ± 0.03	0.025	0.152	
Path length	1.56 ± 0.07	1.57 ± 0.06	1.57 ± 0.06	0.021	0.208	
Efficiency	0.66 ± 0.03	0.65 ± 0.03	0.65 ± 0.03	0.027	0.139	
Low beta band						
Strength	179.14 ± 10.79	175.94 ± 8.94	173.50 ± 7.60	0.083	**0**.**002**	**0**.**001**
Clustering coefficient	0.57 ± 0.03	0.56 ± 0.03	0.55 ± 0.02	0.083	**0**.**002**	**0**.**001**
Path length	1.85 ± 0.09	1.87 ± 0.08	1.90 ± 0.07	0.079	**0**.**002**	**0**.**002**
Efficiency	0.57 ± 0.03	0.56 ± 0.03	0.55 ± 0.02	0.083	**0**.**002**	**0**.**001**
High beta band						
Strength	151.16 ± 14.30	147.59 ± 10.95	145.42 ± 10.81	0.067	**0**.**006**	
Clustering coefficient	0.47 ± 0.04	0.46 ± 0.03	0.45 ± 0.03	0.066	**0**.**007**	
Path length	2.29 ± 0.17	2.33 ± 0.14	2.36 ± 0.15	0.062	**0**.**009**	
Efficiency	0.48 ± 0.05	0.47 ± 0.03	0.46 ± 0.03	0.067	**0**.**006**	
Gamma band						
Strength	134.70 ± 18.02	130.38 ± 14.15	126.69 ± 12.83	0.079	**0**.**002**	**0**.**002**
Clustering coefficient	0.42 ± 0.06	0.40 ±± 0.04	0.39 ± 0.04	0.078	**0**.**003**	**0**.**002**
Path length	2.71 ± 0.29	2.78 ± 0.26	2.84 ± 0.24	0.072	**0**.**004**	
Efficiency	0.43 ± 0.06	0.42 ± 0.05	0.40 ± 0.04	0.080	**0**.**002**	**0**.**002**

^a^*p*-values represent statistically significant differences between the low and high CTQ groups with post-hoc test using Bonferroni correction.

### Nodal level differences in cortical functional networks

Based on significant differences of the low beta and gamma band clustering coefficients among the three groups, we decided to examine possible differences at the local level in the low beta and gamma bands. The nodal clustering coefficient of the high CTQ group was significantly decreased in two nodes for the low beta band (primary somatosensory cortex (BA 1–3): 0.58 ± 0.04 *vs*. 0.55 ± 0.03, *p* < 0.001; middle occipital gyrus (BA 19): 0.57 ± 0.05 *vs*. 0.55 ± 0.04, *p* < 0.001) and one node for the gamma band (superior temporal gyrus (BA 41): 0.46 ± 0.07 *vs*. 0.42 ± 0.05, *p* < 0.001) (Table 3).

**Table 3:** Mean and standard deviation values of clustering coefficients in nodal level for low beta and gamma bands among low, middle, and high childhood trauma questionnaire (CTQ) groups.

	Low CTQ (N = 44)	Middle CTQ (N = 71)	High CTQ (N = 38)	Effect size (η^2^)	*p*	Pairwise test, *p*^a^
	*Mean* ± *SD*					Low vs. High
Low beta band						
Primary somatosensory cortex (BA 1–3)	0.58 ± 0.04	0.56 ± 0.03	0.55 ± 0.03	0.124	<0.001	<0.001
Middle occipital gyrus (BA 19)	0.57 ± 0.05	0.55 ± 0.04	0.55 ± 0.04	0.120	<0.001	<0.001
Gamma band						
Superior temporal gyrus (BA 41)	0.46 ± 0.07	0.44 ± 0.06	0.42 ± 0.05	0.115	<0.001	<0.001

^a^*p*-values represent statistically significant differences between the low and high CTQ groups with post-hoc test using Bonferroni correction.

### Correlation between network indices and psychological characteristics

**Figure 1:**
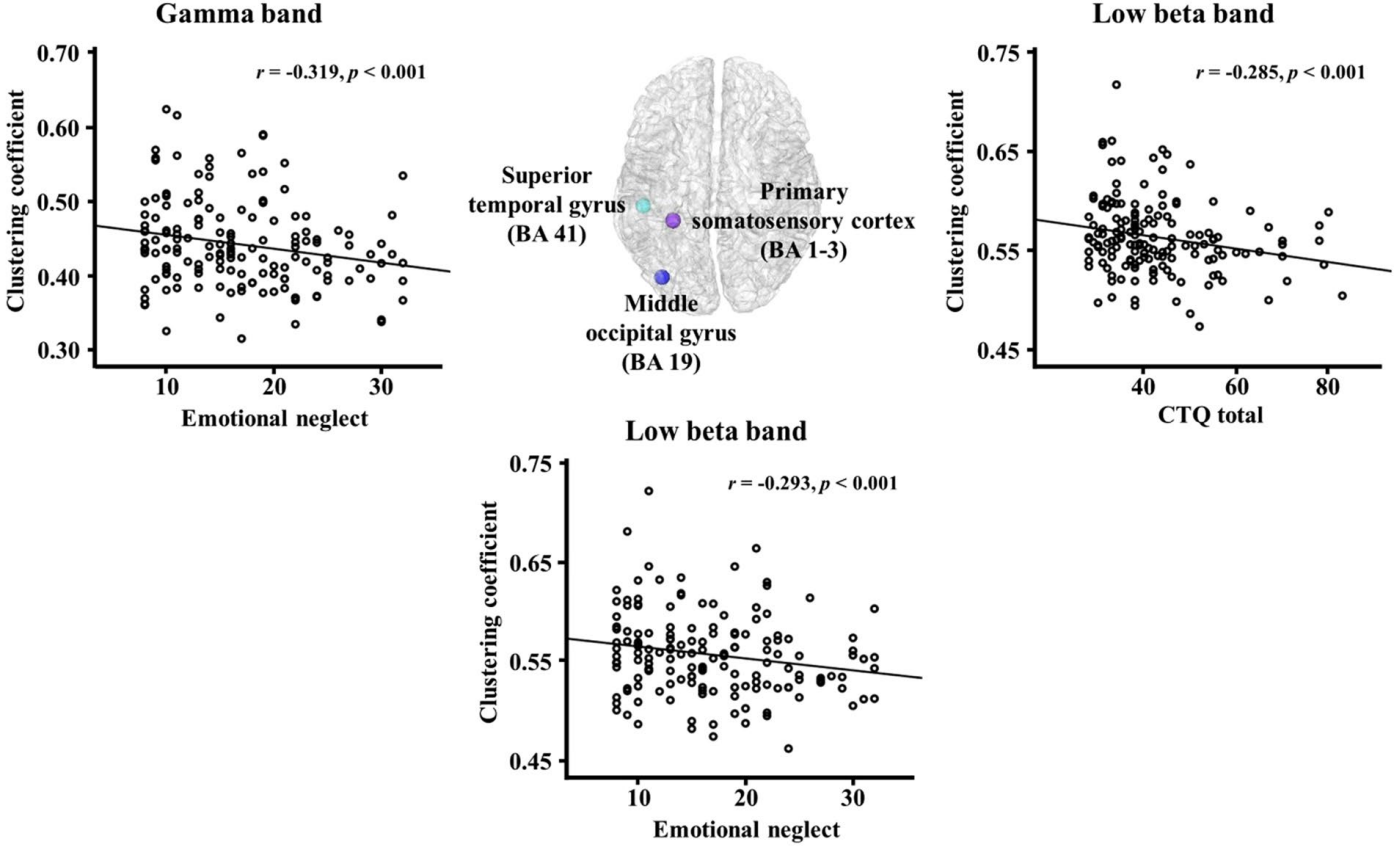
The correlations of the nodal clustering coefficients with childhood trauma-related measures in the low beta and gamma bands. The nodal clustering coefficient at the left primary somatosensory cortex (BA 1–3) showed significant correlations with the childhood trauma questionnaire (CTQ) total score for the low beta band. The nodal clustering coefficients at the left middle occipital gyrus (BA 19) for the low beta band and superior temporal gyrus (BA 41) for the gamma band were significantly correlated with the CTQ emotional neglect subscale score.

The relationships between the network indices at the global and nodal levels and childhood trauma-related measures were investigated in the low beta and gamma bands. The nodal clustering coefficient in the left primary somatosensory cortex (BA 1–3) significantly correlated with the total CTQ score for the low beta band (r = −0.285, *p* < 0.001). In addition, the nodal clustering coefficients in the left middle occipital gyrus (BA 19) for the low beta band and superior temporal gyrus (BA 41) for the gamma band were significantly correlated with emotional neglect (r = −0.293, *p* < 0.001; r = −0.319, *p* < 0.001, respectively) (Fig. 1).

## Discussion

This study evaluated whether individuals with higher CTQ scores show altered cortical functional networks during a behavioral inhibition task (i.e., NoGo-P3 processing of the Go/NoGo task). Our major findings, which pertained to the low beta and gamma bands, can be divided into the global and the nodal levels. First, at the global level, the strength, clustering coefficient, and efficiency of the low beta and gamma bands were significantly decreased in the high CTQ group compared to the low CTQ group. The path length of the low beta band was significantly longer in the high CTQ group than in the low CTQ group. Second, at the nodal level, the clustering coefficient of the low beta band was decreased in the left primary somatosensory cortex and middle occipital gyrus and that of the gamma band was decreased in the left superior temporal gyrus for the high CTQ group. Third, the nodal level low beta band clustering coefficient of the left primary somatosensory cortex was significantly negatively correlated with the total CTQ score. In addition, the nodal clustering coefficient in the left middle occipital gyrus for the low beta band and superior temporal gyrus for the gamma band was significantly negatively correlated with emotional neglect.

In this study, the scores of the STAI, BDI, BIS subscales (attentional impulsivity and motor impulsivity), and CAARS (inattention/memory and hyperactivity/restlessness) were significantly higher in the high CTQ group than in the low CTQ group. This is consistent with the previous literature that has identified significant associations between childhood trauma and the development of depressive and anxiety disorder in adulthood^45,46^, as well as behaviors related to impulsivity^4,47^. Specifically, Brodsky *et al*.^4^ noted that depressed patients with childhood trauma were not only more likely to have attempted suicide but also to have higher BIS scores. Narvaez *et al*.^47^ showed that childhood trauma was strongly associated with poor executive functioning and higher levels of impulsivity among crack cocaine users. Furthermore, Beers and De Bellis revealed that maltreated children with post-traumatic stress disorder showed decreased sustained attention and were more vulnerable to distractions^48^.

In contrast to the STAI, BDI, BIS subscales, and the CAARS, the scores of the Behavioral Activation System (drive) were found to be significantly lower in the high CTQ group. Previously, it has been suggested that childhood maltreatment could alter the sensitivity of the Behavioral Activation System^49^, and also lead to deficits in cognitive control and emotion regulation^2,50^. Our results support the previous findings that individuals with childhood trauma display various psychological and behavioral disruptions.

In this study, no significant differences were reported among the three groups in term of the NoGo false alarm rate. In addition, no significant relationships were found between the network indices and the NoGo false alarm rate. These results are similar to the previous study, which identified no significant correlation between the Go/NoGo task performance and the ratings on childhood trauma^51^. However, studies that have examined the influences of childhood trauma on the Go/NoGo task have not produced entirely consistent results and there have been studies that reported significant associations between childhood trauma and the performance in tasks using the Go/NoGo paradigm. For example, longer reaction times during inhibitory tasks have been reported in individuals with childhood maltreatment^10^. Tottenham *et al*.^50^ reported that institutionalized children, when compared to controls, were more likely to make false alarm errors to negatively valenced faces that appear in the NoGo condition. This suggests that these children may have difficulties regulating emotions.

Although our study did not find any significant effect of childhood trauma on the performance during the Go/NoGo task, we identified apparent differences between the network indices of the low and high CTQ groups. Moreover, the nodal level clustering coefficients showed significant correlations with childhood trauma-related measures during the Go/NoGo task.

The strength, clustering coefficient, and efficiency of the low beta and gamma frequency bands were significantly decreased in the high CTQ group compared to the low CTQ group. On the other hand, the path length of the low beta frequency band was significantly prolonged in the high CTQ group. Decreased clustering coefficient and increased path length could each imply decreased local connectedness and inefficiently increased connectedness of brain networks. Furthermore, decreased strength in the brain networks of the high CTQ group could reflect weaker connection strength (looser links) compared to that of the low CTQ group. Maltreatment during childhood has been associated with changes in the structure and functional connectivity at the network level. Teicher *et al*.^39^ reported that childhood maltreatment was associated with a decrease in the centrality of the left anterior cingulate region and an increase in the centrality of the right anterior insula and precuneus. An fMRI study assessing the inhibitory control network revealed that less inhibition of the right inferior frontal cortex, which was negatively modulated by the dorsal anterior cingulate cortex, improved inhibitory control ability in males with higher CTQ scores^52^. In addition, individuals with childhood trauma showed decreased network indices (degree, efficiency, and betweenness centrality) in the right ventrolateral prefrontal cortex and the dorsal anterior cingulate cortex compared to the control group^38^. The findings from these previous studies support our results that network characteristics are disrupted in individuals with childhood trauma.

Previous studies have reported significant association between altered beta and gamma band activities and childhood trauma. In addition, the presence of beta and gamma bands have been related to symptoms of anxiety and anxiety related disorders^53–55^. Increased beta power is known to occur during sleep in individuals who have experienced childhood maltreatment^56^. Individuals with childhood trauma have demonstrated significantly higher beta coherence over the right central and temporal regions than those with no trauma history^57^. A recent study by our research group showed increased beta and gamma power in individuals with childhood trauma, which suggests that the increased power of individuals with childhood trauma may reflect cognitive deficits of the brain. Furthermore, it revealed that childhood trauma might be associated with high anxiety states that cause the increase of gamma band power in the brain^58^. In addition, increased resting state EEG beta powers in bereaved family members of accident victims have been observed; this increased beta power was interpreted to reflect a coping strategy for extremely complex and stressful situations. The same study also reported that gamma activity was significantly higher in bereaved family members with high anxiety scores^59^. In sum, these findings support the hypothesis that the beta and gamma frequency bands reflect vulnerability to anxiety, stress, and childhood trauma.

In the high CTQ group, the nodal clustering coefficients of the low beta band were decreased in the left primary somatosensory cortex and middle occipital gyrus; in the left superior temporal gyrus, that of the gamma frequency band was decreased. The nodal clustering coefficient of the left primary somatosensory cortex for the low beta band was negatively correlated with the CTQ total score. In addition, the nodal clustering coefficients of the left middle occipital gyrus for the low beta band and the superior temporal gyrus for the gamma band were negatively correlated with the emotional neglect subscale score. Childhood maltreatment has been associated with abnormal development of the sensory systems that relay adverse sensory experiences. For instance, women with childhood maltreatment display thinner left primary somatosensory cortex^60^. In addition, a recent meta-analysis demonstrated that childhood maltreatment was associated decreased primary somatosensory volume^61^. In terms of the left middle occipital gyrus, a recent meta-analysis revealed that individuals with childhood maltreatment had larger gray matter volumes in the region^61^.

In addition, the superior temporal gyrus has been closely associated with childhood trauma. Individuals with childhood maltreatment revealed reduced gray matter volume in the superior temporal gyrus^61^. Another study suggested that childhood maltreatment was associated with altered symmetry in the superior temporal gyrus^62^. Fisher *et al*.^63^ reported that when incorrect NoGo trials were compared to the resting baseline period, foster children who had been maltreated showed significantly stronger activation than non-maltreated children in a number of brain regions, including the left superior temporal gyrus. Furthermore, given that the superior temporal gyri develops relatively late^64,65^, the region may be more susceptible to impairment in individuals with childhood trauma. Taken together, these regions are highly related to childhood trauma and our results support these previous findings.

The clustering coefficient represents how strongly each node is connected with its neighbors, and provides information about the level of local connectedness within a network. A higher clustering coefficient represents higher local efficiency of information transfer^66,67^, while a lower clustering coefficient represents loose coupling and a rapid shift towards network randomness^68,69^. Therefore, reduced clustering coefficient in the left primary somatosensory cortex, the middle occipital gyrus, and the superior temporal gyrus indicates that the regions became less connected with its neighbors in accordance with the severity of childhood trauma.

The limitations of this study were as follows. First, the present study did not use a structured clinical interview to screen possible psychiatric illnesses. Second, although the CTQ has been widely used in research for clinical and non-clinical individuals^70^, the CTQ may not precisely reflect the individual’s traumatic childhood experiences because of its retrospective nature. Third, our results may not extrapolate to clinical individuals.

Despite these limitations, our results are meaningful considering that our study was the first to analyze the source-level small-world network in individuals with childhood trauma using an inhibitory task. Our results demonstrate that the functional clustering of the left primary somatosensory cortex, the middle occipital gyrus, and the superior temporal gyrus was vulnerable and dysfunctional in individuals with childhood trauma, especially in those who had experienced emotional neglect. In addition, our results suggest that source-level network analysis of EEG signals might be used as correlates in individuals with childhood trauma.

## Methods

### Participants

The study was performed on 153 non-smoking non-clinical volunteers (56 males and 97 females) with a mean age of 27.75 ± 6.43 years. Participants were recruited from the local community through newspapers and posters. Participants, who revealed any history of neurological or psychological diseases during the initial screening interview, were excluded from the study. All participants had normal or corrected-to-normal vision, as determined by a check of visual acuity with the Snellen chart^71^. The participants were divided into 3 subgroups based on the 25% and 75% quartiles of the Childhood Trauma Questionnaire (CTQ)^72^ total score (34.0 and 47.5, respectively): low CTQ group (lower 25%, n = 44, 31.41 ± 2.06), middle CTQ group (25–75%, n = 71, 40.44 ± 3.61), and high CTQ group (upper 25%, n = 38, 60.21 ± 10.00). The Institutional Review Board at Inje University Ilsan Paik Hospital approved the study and all of its experimental protocols (2015-07-026-001). The study was performed in accordance with approved guidelines and an informed consent was obtained from all individuals included in the study.

### Psychological measures

Anxiety and depression was measured through the State-Trait Anxiety Inventory (STAI)^73,74^ and the Beck Depression Inventory (BDI)^75^. The STAI is a self-rating scale of state and trait anxiety^74^. It consists of a state anxiety inventory (SAI) and a trait anxiety inventory (TAI), which are comprised of 20 items^73^. The BDI is a self-rating scale composed of 21 items that measures the severity of symptoms related to depression^75^.

The Barratt Impulsiveness Scale (BIS)^76,77^ and Conners’ Adult ADHD rating scale (CAARS)^78^ were used to assess impulsivity-related traits. The BIS consists of 30 items, and is designed to assess the personality/behavioral construct of impulsiveness. It is consisted of three sub-factors: attentional, motor, and non-planning impulsivity^76^. The CAARS is designed to assess manifestations of ADHD in adults, and is composed of 42 items that are divided into four subscales: inattention/memory, hyperactivity/restlessness, impulsivity/emotional lability, and problems with self-concept^78^.

Behavioral Inhibition System and Behavioral Activation System scales were used to measure the self-reported dysregulations of behavioral inhibition and activation. The Behavioral Inhibition System and Behavioral Activation System scales are 20-item self-rating scales with good psychometric properties^79,80^. The scales assess the Behavioral Inhibition System (7 items) and three subdomains of the Behavioral Activation System: Drive (4 items), Fun-Seeking (4 items), and Reward Responsiveness (5 items).

The CTQ^72^ was used to assess traumatic childhood experiences. The CTQ consists of 28 items (25 clinical and 3 validity items) that measure 5 categories of childhood maltreatment, including physical, emotional, and sexual abuse, as well as physical and emotional neglect. Each subscale has 5 items with a 5-point frequency of occurrence ranging from 5 to 25.

### Recording and Preprocessing of electroencephalography (EEG)

EEG was recorded using a NeuroScan SynAmps amplifier (Compumedics USA, Charlotte, NC) with 64 Ag-AgCl electrodes mounted on a Quik-Cap using an extended 10–20 placement scheme. The ground electrode was placed on the forehead and the physically linked reference electrode was attached to both mastoids. The vertical electrooculogram (EOG) channels were positioned above and below the left eye, and the horizontal EOG channels were recorded at the outer canthus of each eye. The impedance was maintained below 5 kΩ. All data were processed with a 0.1–100 Hz band pass filter and sampled at 1000 Hz.

The recorded EEG data were preprocessed using CURRY 7 (Compumedics USA, Charlotte, NC). Gross artifacts such as movement artifacts were rejected by visual inspection by a trained individual with no prior information regarding the origin of the data. Artifacts related to eye movement or eye blinks were removed using the mathematical procedure^81^ implemented in the preprocessing software of CURRY 7. The data were filtered using a 0.1–55 Hz bandpass filter and epoched from 100 ms pre-stimulus to 900 ms post-stimulus. The epochs were rejected from further analysis if they contained significant physiological artifacts (amplitude exceeding ± 75 μV) at any site over the 62 electrodes. For the analysis of the Go/NoGo task, only epochs corresponding to correct responses were used. The number of Go/NoGo epochs used for the analysis did not significantly differ among the low, middle, and high CTQ groups (Go condition: 207.16 ± 22.61 *vs*. 202.15 ± 26.36 *vs*. 201.76 ± 24.77, *p* = 0.514, NoGo condition: 48.18 ± 7.43 *vs*. 47.27 ± 6.77 *vs*. 47.29 ± 7.98, *p* = 0.784).

#### Go/NoGo experiment

Participants were seated approximately 60 cm away from a computer screen (Mitsubishi, 22-inch CRT monitor). Stimuli for the Go/NoGo task, which consisted of the numbers 1–8, were presented randomly on the screen. The participants were instructed to press a space bar as accurately and quickly as possible when the Go stimuli (even numbers: 2, 4, 6, and 8) appeared at the center of the screen and to not respond when the NoGo stimuli (odd numbers: 1, 3, 5, and 7) were displayed. There were 300 trials comprising the Go (80% probability) condition and the NoGo (20% probability) condition. For each trial, a fixation cross was presented for 100 ms. Following intervals of 700–1000 ms, the Go or NoGo targets appeared for 500 ms. In between trials, there was a 500 ms interval. These stimuli were generated by E-Prime software (Psychology Software Tools, Pittsburgh, PA, USA).

### Source localization

The minimum-norm estimation, which was implemented in eConnectome toolbox (Biomedical Functional Imaging and Neuroengineering Laboratory, University of Minnesota, Minneapolis, MN), was used to estimate the time series of source activities^82^. A three-layer boundary element method (BEM) model, constructed from the MNI 152 standard template, was used to compute the lead field matrix. Cortical current density values at 7,850 cortical vertices were evaluated for every time point of each epoch. After estimating the cortical current density at every time point, 314 nodes were extracted as evenly as possible from the original cortical surface model^69^. The time series of the cortical sources at each of the 314 nodes were bandpass filtered and divided into four frequency bands: alpha (8–12 Hz), low beta (12–18 Hz), high beta (18–30 Hz), and gamma (30–55 Hz). Lower frequency bands such as delta (1–4 Hz) and theta (4–8 Hz) were not considered in the analysis because only one or two full cycles of these frequency components were included in the analysis time window (500 ms), and inclusion may have caused significant bias in the phase-locking analysis results. The time interval, which was based on previous studies and included the P300 component, was set from 200 to 700 ms after the NoGo target stimulus onset^21,83^. Visual inspection of the grand-averaged waveforms at the 4 electrodes of interest (Fz, FCz, Cz, and Pz), all of which were located on the midline^84^, was performed.

### Connectivity and network analysis

The functional connectivity between each pair of nodes was evaluated using phase-locking values (PLVs)^85^. PLVs were used as the measure of synchronization because they range from 0 to 1, and can be directly used to represent the connection strength in the weighted network analysis without further modifications.

In this study, we performed weighted network analysis based on the graph theory^35,36^. The use of weighted networks is not only free from ambiguity in determining the threshold values, but can also preserve the unique traits of the original network without distortion. A network is composed of several nodes that are connected to each other at their edges. Four different global level weighted network indices were evaluated. First, ‘strength’ refers to the degree of connection strength in the network. It is estimated by summing up the weight of links connected to the brain regions. Second, ‘clustering coefficient’ indicates the degree of which a node is clustered with its neighboring nodes. Clustering coefficient was calculated for the whole network. Third, ‘path length’ indicates overall connectedness of the whole network and is calculated as the sum of lengths between two nodes in the entire network. Fourth, ‘efficiency’ refers to the efficiency of information processing in the brain. Additionally, the weighted nodal clustering coefficient was evaluated for each node (supplementary).

### Statistical analysis

A one-way analysis of variance (ANOVA) was conducted to compare the scores of psychological and behavioral data among the three groups. In addition, a multivariate ANOVA (MANOVA) was conducted to compare the cortical network characteristics at the global level for each frequency band among the three groups with STAI, BDI, and CAARS as covariates. The multiple comparison problems were solved through the Bonferroni correction with an adjusted *p*-value of 0.05/16 = 0.003125. The identical analysis was conducted at the nodal level, followed by the Bonferroni correction with an adjusted *p*-value of 0.05/314 = 0.000159. The variables showing significant differences among three groups were further analyzed using post hoc pairwise comparisons, namely the Bonferroni correction. Effect sizes were expressed as partial eta squared (η^2^). In addition, the relationships between network indices and childhood trauma-related measures were analyzed by a partial Pearson’s correlation in which STAI, BDI, and CAARS were controlled for. The correlation analysis was followed by the Bonferroni correction with an adjusted *p*-value of 0.05/60 = 0.000833. Statistical analyses were performed using SPSS 21 (SPSS, Inc., Chicago, IL). The power of cortical network characteristic differences at the global level among the three groups was calculated using the G*Power 3.1.9 software^86^.

## Electronic supplementary material

Supplementary Material
