# Supplementary material for: Altered cortical functional network during behavioral inhibition in individuals with childhood trauma
Source: Sci Rep. 2018 Jul 4;8:10123. doi: 10.1038/s41598-018-28329-6 (PMC6031680; doi:10.1038/s41598-018-28329-6)
Supplement: Supplementary file 1 — Supplementary Material [file 41598_2018_28329_MOESM1_ESM.docx]

**Altered cortical functional network during behavioral inhibition in individuals with childhood trauma**

**Sungkean Kim^1,2^, Ji Sun Kim^3^, Miseon Shim^4^, Chang-Hwan Im^2^, Seung-Hwan Lee^1,5^**

^1^Clinical Emotion and Cognition Research Laboratory, Inje University, Goyang, Republic of Korea

^2^Department of Biomedical Engineering, Hanyang University, Seoul, Republic of Korea

^3^Department of Psychiatry, Soonchunhyang University Cheonan Hospital, Cheonan, Republic of Korea

^4^Department of Psychiatry, University of Missouri-Kansas City, Kansas City, Missouri, USA

^5^Department of Psychiatry, Inje University, Ilsan-Paik Hospital, Goyang, Republic of Korea

**Address correspondence and reprint requests to:**

Seung-Hwan Lee, MD, PhD

Department of Psychiatry, Ilsan Paik Hospital, Inje University College of Medicine, Juhwa-ro 170, Ilsanseo-Gu, Goyang, 411-706, Korea.

E-mail: lshpss@paik.ac.kr, lshpss@hanmail.net (Tel.) +82-31-910-7260, (Fax) +82-31-910-7268

**Supplementary Material**

**Network indices**

The weighted network was quantitatively analyzed based on the graph theory. Four different global level weighted network indices were evaluated:

1. Strength: the degree of connection strength in the network. It is estimated by summing up the weight of links connected to the brain regions.

2. Clustering coefficient: how strongly each node is connected with its neighbors. Clustering coefficient quantified the degree of which a node is clustered with its neighboring nodes. Clustering coefficient *C* in each node is defined by

$$C_{i}= \frac{1}{K_{i}\left( K_{i}-1 \right)}\sum_{j,k\in G, j, k\neq i} \left( w_{ij}\cdot w_{jk}\cdot w_{ki} \right)^{\frac{1}{3}}$$

where *K* is the number of neighbors of a vertex and *w* is a weight of links connected to the brain regions. Global clustering coefficient is defined as the average of the local clustering coefficients of all the vertices by

$$\bar{C}=\frac{1}{n}\sum_{i=1}^{n} C_{i}$$

3. Path length: how well the nodes communicate with each other. It is estimated as the sum of lengths between two nodes in the entire network. Path length L is defined by

$$L= \frac{1}{N\left( N-1 \right)}\sum_{i, j\in G, i\neq j} d_{ij}$$

where *N* is the number of nodes, *d* is the shortest distance between node *i* and *j*, and *d_ij_* is defined by

$$d_{ij}=\min\left( \frac{1}{w_{ji}}+\cdots+\frac{1}{w_{hj}} \right)$$

4. Global efficiency: the efficiency of information processing in the brain. Global efficiency is defined by

$$E_{global}=\frac{1}{N\left( N-1 \right)}\sum_{j\in G} \frac{1}{d_{ij}}$$

Additionally, the weighted nodal clustering coefficient was evaluated for each node.
